# Seasonal Variation of Airborne Bacteria and Potential Pathogens Adhere to Fine and Coarse Particles in a Coastal City

**DOI:** 10.3390/microorganisms14091924

**Published:** 2026-08-31

**Authors:** Yunhan Qin, Yidan Tian, Tingfu Li, Changliang Nie, Xueyun Geng, Xiaomin Sun, Lingyu Li, Yoshizumi Kajii

**Affiliations:** 1 Shandong Key Laboratory of Synergistic Control of Complex Multi-Media Pollution, School of Environment and Geography Science, Qingdao University, Qingdao 266071, Chinalilingyu@qdu.edu.cn (L.L.); kajii.yoshizumi.7e@kyoto-u.jp (Y.K.); 2 Environment Research Institute, Shandong University, Qingdao 266237, China; 3 State Key Laboratory of Microbial Technology, Shandong University, Qingdao 266237, China

**Keywords:** bacteria, bioaerosols, fine particles, coarse particles, season

## Abstract

Airborne bacteria exhibit high taxonomic diversity and a well-documented capacity to influence human health through pathogenic infection, allergenic sensitization, and immunomodulatory or commensal exposure. However, characterization of the airborne bacterial community structure, and particularly the seasonal dynamics, size-resolved distribution, and functional potential of predicted potential pathogens in Qingdao, a coastal megacity on the Yellow Sea, remains limited. Results revealed pronounced seasonal variation in community structure: *Staphylococcus*, *Lactobacillus*, *Escherichia*–*Shigella*, and *Klebsiella* were consistently dominant across all samples. LEfSe analysis identified *Staphylococcus* as significantly enriched in autumn, supporting its utility as a robust seasonal biomarker (LDA > 3.5, *p* < 0.05). This genus is predominantly associated with human sources and comprises opportunistic pathogens, thereby posing a potential risk to human health. Significant compositional divergence between summer and autumn communities was observed as a higher beta deviation index, whereas there were no statistically significant differences between particle size groups. The Chao and Shannon indices were consistently higher in PM_10_ than in PM_2.5_ across all seasons; however, this size-associated difference reached statistical significance only for autumn PM_10_ (*p* < 0.05), indicating a seasonally amplified effect of coarse particulate matter on microbial richness and evenness. These analyses provide new insight into the total airborne bacterial and potential pathogens in an urban coastal environment.

## 1. Introduction

Bioaerosols, comprising bacteria, fungi, viruses, and other biological particles, can influence climate processes and pose potential threats to human health [1]. For instance, airborne fungi on particulate matter pose health risks including respiratory infections, allergies, and invasive disease in vulnerable individuals [2,3]. COVID-19 is another example of airborne disease, causing widespread harm to human health [4,5]. Among the bioaerosol components, airborne bacteria represent one of the most significant groups [6] owing to their high taxonomic diversity and well-documented potential to impact human health [6,7] and interactions with terrestrial microbiomes and anthropogenic activities [8]. Consequently, airborne bacteria have attracted increasing scientific attention over recent years [5]. Both total bioaerosols and specific potential pathogens exhibit distinct environmental origins [9,10,11]: aquatic bacteria are frequently emitted via bubble-burst mechanisms at the ocean or freshwater surface [12]; soil-associated bacteria are resuspended during agricultural activities [13]; oil-related bacteria are enriched in emissions from cooking [14]; and animal-associated taxa are commonly elevated near livestock farming operations [15]. Furthermore, geographic location, including city-specific characteristics, and atmospheric conditions such as haze episodes exert selective influences on airborne bacterial community composition [10,16]. For instance, distinct regional signatures have been observed across Chinese cities: southern coastal and inland cities, including Shanghai [17], Guangzhou [18,19], and Nanjing [20], exhibiting different community profiles compared with northern inland cities such as Beijing [21,22], Xi’an [23], and Jinan [24]. Notably, *Anoxybacillus*, *Brevibacillus*, and *Acinetobacter* were identified as the dominant genera in Guangzhou [18], whereas *Staphylococcus* predominated in Jinan [24]. These spatially structured patterns underscore the necessity of city-specific investigations to accurately characterize local bioaerosol sources, dynamics, and health implications.

Qingdao, a coastal megacity situated on the Yellow Sea, has attracted substantial scientific attention due to its unique geographic setting, directly adjacent to the ocean, and consequent strong marine influence. Previous studies have characterized airborne bacteria in this region [25]. For instance, Wei et al. (2022) investigated inhalable bioaerosol composition and deposition efficiency in the human respiratory tract under foggy and hazy conditions [26]. Yin et al. (2021) reported average total airborne microbial concentrations of 6.75 × 10^5^ cells/m^3^ on haze days and 1.03 × 10^6^ cells/m^3^ on dust days [27]. Seasonal analyses further revealed significant variation in alpha diversity indices: species richness, evenness, and Shannon diversity were highest in summer, followed by autumn and spring, and were lowest in winter [25]. Consistent with this pattern, microbial metabolic activity was also elevated in summer and autumn relative to other seasons [28]. Moreover, atmospheric deposition serves as a recognized pathway for the input of airborne microbes into the marine environment, potentially influencing nutrient cycling, microbial community assembly, and biogeochemical processes in coastal waters [29]. Despite these advances, comprehensive characterization of the airborne bacterial community structure, and particularly the seasonal dynamics and distribution of potential pathogens, remain limited.

Several studies have comparatively characterized the composition and structure of total bacterial communities versus potential pathogen subsets across PM size fractions. Notably, bacterial alpha diversity and the relative abundance of potential pathogens differ significantly among PM_10_, PM_2.5_, and total suspended particles (TSP), reflecting size-selective enrichment and differential atmospheric processing [30]. Pathogenic bacteria often exhibit enhanced environmental resilience, including resistance to UV radiation, desiccation, and oxidative stress, compared with non-pathogenic taxa, facilitating their survival during long-range atmospheric transport [31]. Regarding the particle size, PM_10_ (aerodynamic diameter ≤10 μm) is primarily deposited in the upper respiratory tract and associated with cytotoxicity, mucosal inflammation, and impaired mucociliary clearance [32]. PM_2.5_ (≤2.5 μm) penetrates deeply into the alveolar region and can translocate across the alveolar–capillary barrier into systemic circulation, contributing to both respiratory and cardiovascular pathologies [33]. To our knowledge, few previous studies have focused on the influence of seasonality and particle size effects on airborne total and potential pathogenic bacteria in Qingdao, so the objective of this study was focused on the sequencing of PM_2.5_ and PM_10_ to resolve the taxonomic and community profiles of total bacteria and potential pathogens. These analyses provide mechanistic insight into the atmospheric fate, transport drivers, and public health relevance of airborne bacterial pathogens in an urban coastal setting.

## 2. Methods and Materials

### 2.1. Sample Collection and Pretreatment

Atmospheric particulate matter (PM) samples were collected in Qingdao, a coastal city in eastern China characterized by a temperate monsoon climate with marked seasonal variation in meteorological conditions and air quality. Samples were conducted during two distinct periods: August (summer) and November (autumn) 2021, as shown in Table S1, using a mid-volume air sampler (TH-150A, Wuhan Tianhong Instruments Co., Ltd., Wuhan, China) according to a previous study [13]. Equipped with certified PM_2.5_ and PM_10_ size-selective inlets on the roof of a single-story coastal building with unobstructed exposure in all directions, the sampling site provides an ideal setting for atmospheric aerosol collection. Sampling was performed at a constant flow rate of 100 L/min, with each sample collected continuously for 23 h onto prebaked 88-mm-diameter quartz fiber filters (Whatman QMA, Clifton, NJ, USA). Prior to deployment, the air flow of the sampler was calibrated, and all filters were combusted in a muffle furnace at 450 °C for 4.5 h to remove residual organic carbon and ensure baseline purity. Blank filters were prepared by inserting sterile quartz filters into the sampler without actuating it; subsequent DNA extraction and PCR yielded no amplification, confirming the absence of prior contamination. Sample identifiers followed a standardized nomenclature: “F” denoted PM_2.5_ filters, and “C” denoted PM_10_ filters; the digit number appended to each letter indicates the sampling date. A detailed group-stratified analysis is presented in Table S2 and further elaborated in the Supplementary Materials.

Upon the completion of sampling, particulate-laden quartz fiber filters were aseptically retrieved using sterile stainless-steel forceps, immediately sealed in individually labeled sterile polyethylene bags, and transported on ice to the laboratory within 2 h. All samples were stored at −20 °C in ultra-low-temperature freezers until DNA extraction and downstream analysis to preserve microbial community integrity and prevent nucleic acid degradation.

### 2.2. DNA Extraction and High-Throughput Sequencing

Each quartz fiber filter was aseptically divided into small sections; genomic DNA was extracted using the TIANGEN Soil DNA Kit (Cat. No. DP328, Beijing, China) following the manufacturer’s protocol. DNA concentration and purity were assessed by spectrophotometry using a NanoDrop^®^ ND-2000 instrument (Thermo Fisher Scientific, Waltham, MA, USA). Extracted DNA was stored at −20 °C until sequencing. For high-throughput amplicon sequencing, the V3–V4 hypervariable region of the bacterial 16S rRNA gene was amplified by PCR using the forward primer 338F (5′-ACTCCTACGGGAGGCAGCAG-3′) and reverse primer 806R (5′-GGACTACHVGGGTWTCTAAT-3′). Triplicate 30-μL reactions were performed per sample on an ABI GeneAmp^®^ 9700 thermal cycler (Applied Biosystems, Foster City, CA, USA), each containing 15 μL 2× Phanta^®^ Max Master Mix, 0.6 μL dNTP mixture (10 mM each), 2.4 μL combined primers (10 μM each), 0.6 μL Phanta^®^ Max Super-Fidelity DNA Polymerase, template DNA, and nuclease-free water to volume. Thermal cycling conditions consisted of initial denaturation at 98 °C for 3 min; 35 cycles of denaturation at 98 °C for 10 s, annealing at 55 °C for 30 s, and extension at 72 °C for 45 s; and a final extension at 72 °C for 10 min.

All PCR amplifications included sterile nuclease-free water as a negative control to monitor for reagent contamination. Amplified products from triplicate reactions per sample were pooled, resolved by 2% (*w*/*v*) agarose gel electrophoresis, and were purified using the AxyPrep DNA Gel Extraction Kit (Axygen Biosciences, Union City, CA, USA) following the manufacturer’s instructions. Purified amplicons were quantified fluorometrically using a Quantus™ Fluorometer (Promega, Madison, WI, USA) with Qubit™ dsDNA HS Assay reagents. Aliquots of purified DNA were stored at −80 °C until library construction. Sequencing libraries were prepared from normalized amplicon pools using the Illumina TruSeq™ DNA PCR-Free Library Preparation Kit (Illumina, San Diego, CA, USA), and sequenced on an Illumina MiSeq platform in paired-end mode (2 × 300 bp) at Majorbio Bio-Pharm Technology Co., Ltd. (Shanghai, China).

### 2.3. Data Analysis

Bioinformatics analysis of particulate matter data was performed using the Majorbio Cloud platform (https://cloud.majorbio.com, accessed on 24 August 2026). Based on ASV information, α-diversity metrics including Chao richness and Shannon index were calculated using Mothur v1.30.1 software. Wilcoxon’s and Kruskal–Wallis tests were performed, and the difference was considered significant at *p* < 0.05. Microbial community similarity between samples was assessed via non-metric multidimensional scaling (NMDS) based on Bray–Curtis dissimilarity, performed using the Vegan v 2.5-3 software package. Linear discriminant analysis effect size (LEfSe) analysis was conducted to identify specific taxa among different sample groups. The Kruskal–Wallis sum-rank test was performed to examine the changes and dissimilarities among classes, followed by LDA (LDA > 3.5 and *p* < 0.05) analysis to determine the size effect of each distinctively abundant taxon. BugBase predicted the bacterial ecological and metabolic functions according to previous description [14]. BugBase is a computational tool designed to predict microbial phenotypes from 16S rRNA gene amplicon sequencing data derived from environmental samples. It first normalizes operational taxonomic units (ASVs) by estimating 16S rRNA gene copy numbers, thereby correcting for biases introduced by variable copy number across taxa. Subsequently, it infers phenotypic traits, including Gram staining classification (Gram-positive or Gram-negative), pathogenic potential, biofilm formation capacity, motility, and oxygen requirement, using pre-computed phenotype profiles mapped to reference taxa [34]. Based on bacterial phenotype prediction, we extracted the top 50 genera in the table corresponding to potentially pathogenic taxa for potential pathogen analysis.

## 3. Results

### 3.1. The Total Bacteria and Potential Pathogens Community in PM_2.5_ and PM_10_

**Figure 1:**
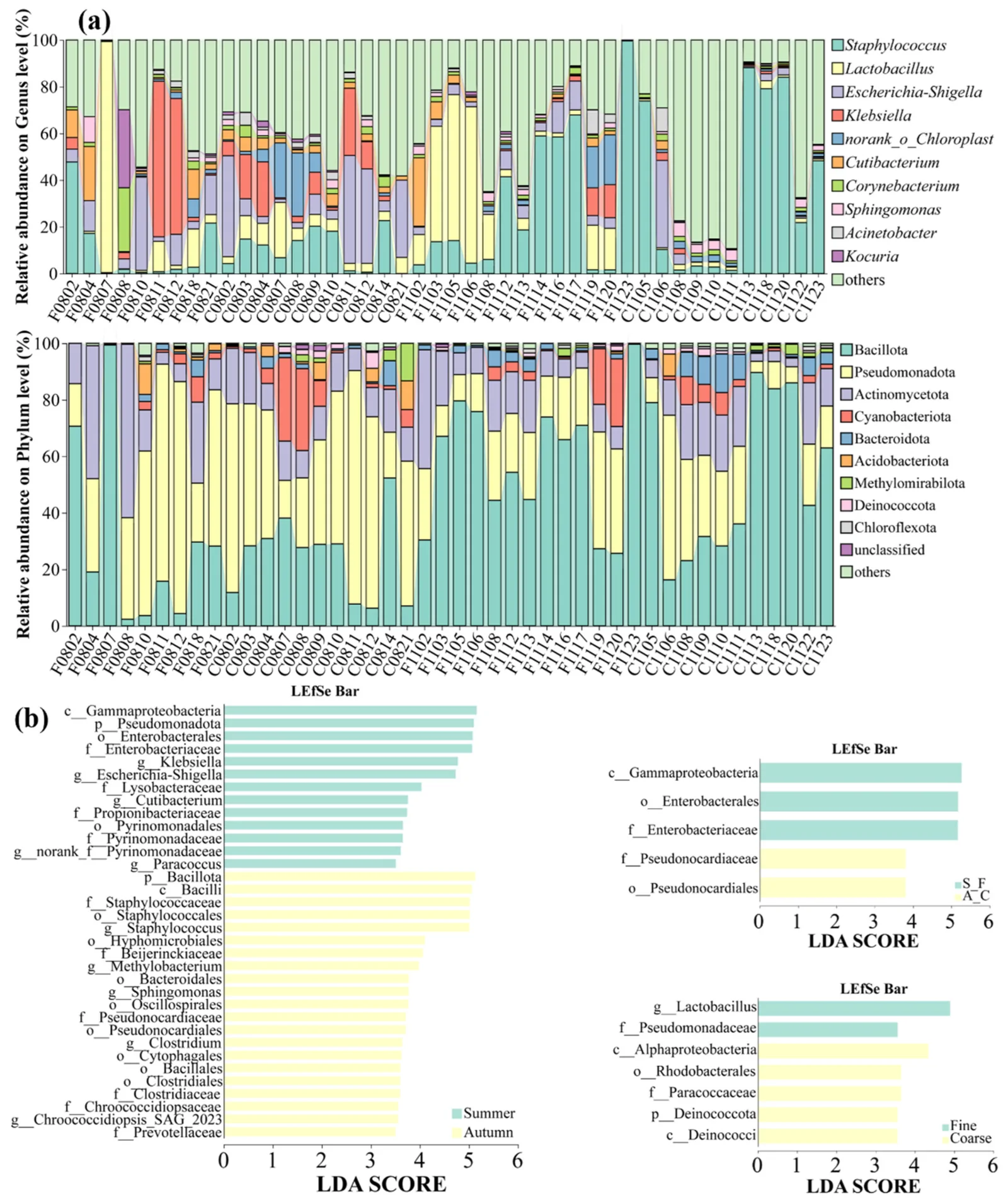
The bacterial community in PM_2.5_ and PM_10_. The bacterial community across all samples at the genus and phylum levels (**a**). The LEfSe analysis identified the biomarkers in different groups (**b**).

The relative abundances of the top 10 genera are shown in Figure 1a. LEfSe analysis further identified taxonomic biomarkers significantly enriched in specific sample groups (Figure 1b). At the genus level, *Staphylococcus*, *Lactobacillus*, *Escherichia*–*Shigella*, and *Klebsiella* were consistently among the most abundant taxa across all samples.

However, their relative abundances exhibited pronounced seasonal and size-fraction-dependent variation. *Staphylococcus* was significantly enriched in autumn (mean relative abundance: 30.32%) compared with summer (7.74%), a pattern corroborated by LEfSe analysis identifying it as a statistically robust seasonal biomarker (LDA > 3.5, *p* < 0.05). Moreover, *Staphylococcus* showed a clear particle-size stratification: its mean abundance was higher in PM_10_ (24.03%) than in PM_2.5_ (17.18%) overall; this size-associated enrichment intensified seasonally, rising from 10.20% in summer PM_10_ to 36.27% in autumn PM_10_, and from 4.04% in summer PM_2.5_ to 24.61% in autumn PM_2.5_. However, the difference was not statistically significant; thus, *Staphylococcus* was excluded from biomarker identification across particle size groups.

*Klebsiella* was identified as a statistically significant seasonal biomarker for summer, with a mean relative abundance of 11.54%, nearly sixfold higher than in autumn (1.79%). This summer enrichment was further stratified by particle size: *Klebsiella* accounted for 14.99% of the bacterial community in summer PM_2.5_, declining to 9.25% in summer PM_10_; in autumn, its abundance dropped sharply to 2.78% in PM_2.5_ and 0.77% in PM_10_. *Lactobacillus* was significantly enriched in PM_2.5_ relative to PM_10_ (mean: 22.72% vs. 4.46%), and thus emerged as a size-fraction-specific biomarker for fine particles. Its abundance also exhibited modest seasonal variation, representing 15.39% of the community in summer and 11.14% in autumn. *Escherichia*–*Shigella* displayed a similar seasonal pattern, contributing 14.37% of the bacterial composition in summer and declining to 4.88% in autumn.

At the phylum level, Bacillota, Pseudomonadota, Actinomycetota, and Cyanobacteriota were the four most abundant bacterial phyla across all samples. Bacillota exhibited the highest relative abundance, ranging from 2.35% to 99.69%; Pseudomonadota ranged from 0.12% to 82.62%; and Actinomycetota ranged from 0.02% to 61.39%. Cyanobacteriota, while consistently present, showed lower and more variable abundance. Several phyla exhibited significant group-specific differences. For instance, Pseudomonadota showed pronounced seasonal variation and was identified as a summer-enriched biomarker (LDA score > 3.5, *p* < 0.05); Bacillota was significantly enriched in the autumn group (LDA score > 3.5, *p* < 0.05).

We further characterized the airborne bacterial community at the species level, as shown in Figure S1. The analysis revealed that a substantial proportion of ASVs were assigned to unclassified species-level taxa. To improve species-level identification in future studies, we plan to integrate culture-dependent isolation with shotgun metagenomic sequencing.

**Figure 2:**
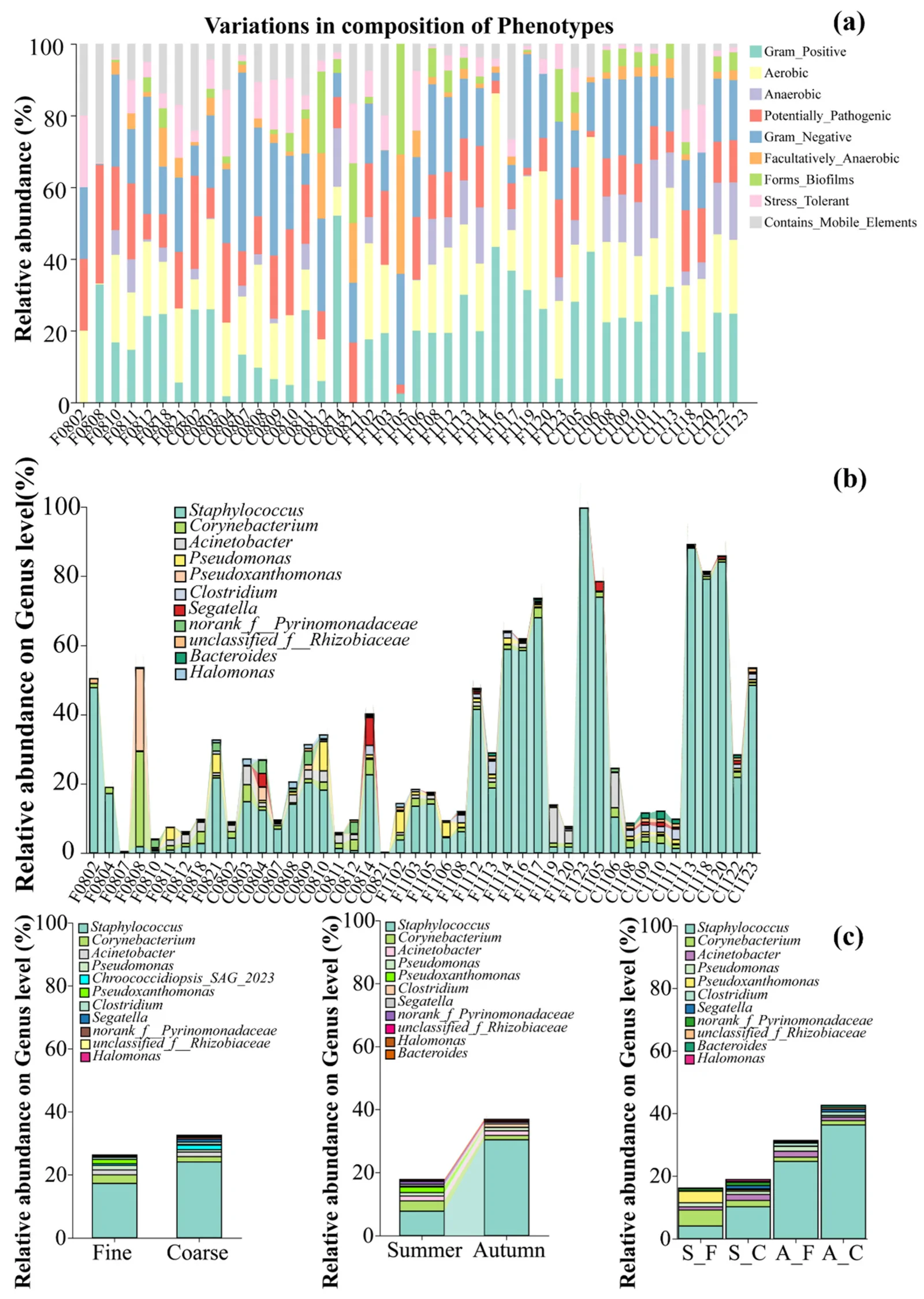
The variations in the composition of predicted phenotypes based on BugBase across all the samples (**a**). The relative abundance of potential pathogens across all samples (**b**). The potential pathogens distribution in different groups (**c**).

To further characterize the functional potential of bacterial communities in PM_2.5_ and PM_10_, BugBase analysis was applied to predict phenotype-level traits. As shown in Figure 2, a substantial proportion of the inferred community was predicted to comprise potential pathogens. The relative abundances of predicted phenotypic traits across all samples ranged as follows: Gram-positive (0–52.05%), aerobic (0–42.84%), potential pathogens (1.66–33.07%), Gram-negative (0.29–49.76%), anaerobic (0–21.77%), harboring mobile genetic elements (0–33.14%), biofilm-forming (0–30.86%), and facultatively anaerobic (0–33.33%).

Potential pathogens were detected across all samples. As shown in Figure 2b, *Staphylococcus* was the most abundant genus among the predicted pathogenic taxa, driving the significantly higher relative abundance of potential pathogens in autumn and in the PM_10_ fraction. However, taxonomic assignment at the species level revealed that most *Staphylococcus* reads were classified only to the genus level, with no confident assignment to known pathogenic species (e.g., *S*. *aureus*, *S*. *epidermidis*). This indicates a critical limitation in current reference database resolution and underscores the need for higher-resolution identification methods, such as full-length 16S rRNA gene sequencing or metagenomic assembly, in future studies.

In addition to *Staphylococcus*, other genera predicted to harbor potential pathogens included *Corynebacterium*, *Acinetobacter*, *Pseudomonas*, *Pseudoxanthomonas*, *Clostridium*, and *Segatella*. Seasonal and size-fraction-specific relative abundances were as follows: *Corynebacterium* exhibited the highest abundance in summer PM_2.5_ (5.10%) and lowest in autumn PM_10_ (1.39%); *Acinetobacter* peaked in summer PM_10_ (1.90%) and declined in autumn PM_10_ (1.09%); *Pseudomonas* showed modest enrichment in autumn PM_2.5_ (1.54%) but reduced representation in autumn PM_10_ (0.52%); *Pseudoxanthomonas* was predominantly detected in summer PM_2.5_ (3.68%), with sharp declines in summer PM_10_ (0.46%) and autumn PM_2.5_ (0.10%); *Clostridium* increased across both seasons and fractions, rising from 0.15% (summer PM_2.5_) to 1.14% (autumn PM_10_); and *Segatella*, nearly absent in summer PM_2.5_, was consistently enriched in coarse particles, reaching 1.00% in summer PM_10_ and 0.78% in autumn PM_10_.

### 3.2. The α Diversity of Airborne Bacteria and Potential Pathogens

**Figure 3:**
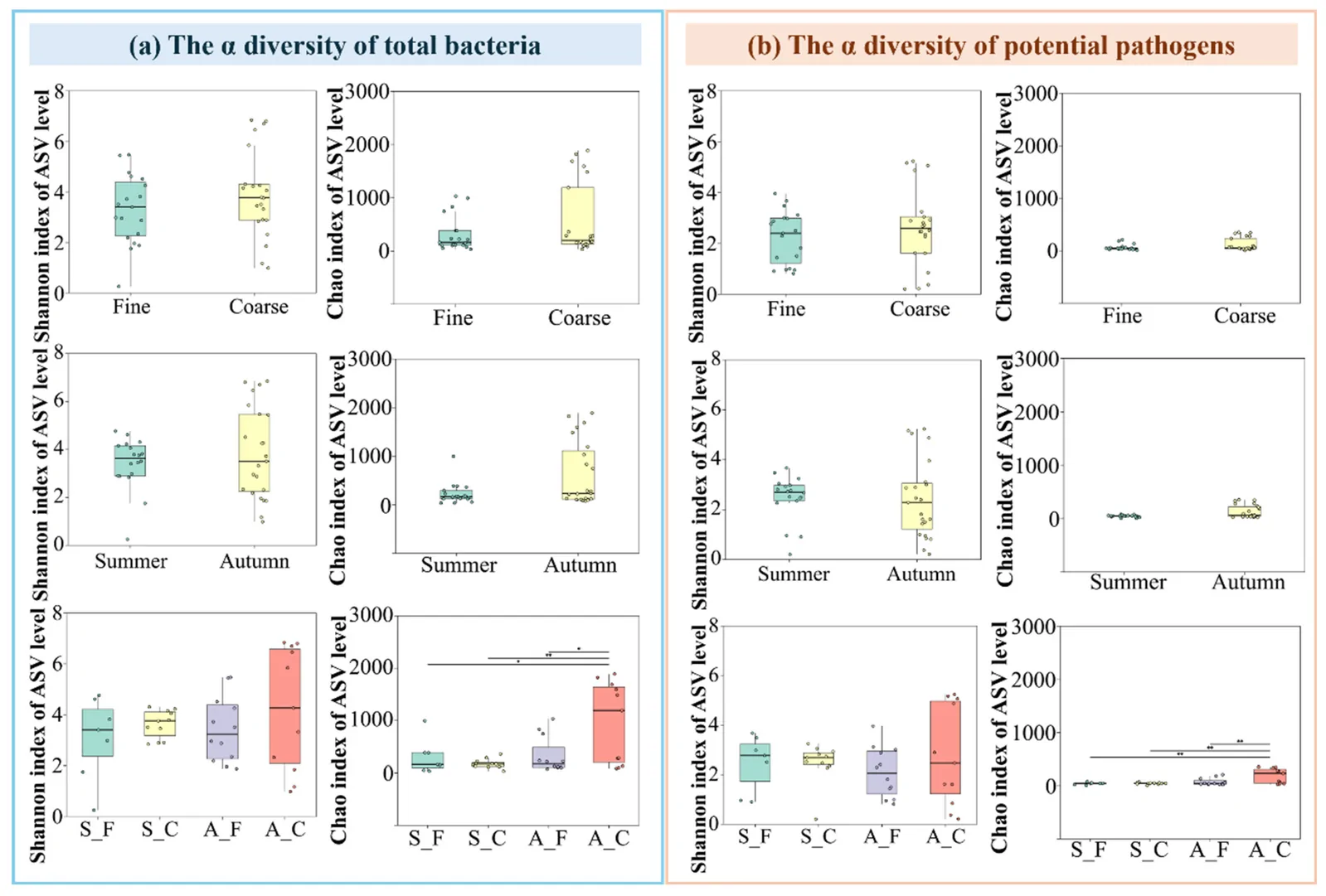
The α diversity, including Shannon and Chao indices of the total bacteria (**a**) and potential pathogens (**b**). (* 0.01 < *p* ≤ 0.05, ** 0.001 < *p* ≤ 0.01).

As shown in Figure 3, α-diversity indices (Shannon and Chao) revealed no statistically significant differences across seasonal groups (summer vs. autumn) or particle size fractions (PM_2.5_ vs. PM_10_) overall (*p* > 0.05). However, the PM_10_ fraction collected in autumn exhibited a significantly higher Chao index than all other groups (*p* < 0.05), suggesting a comparatively richer and more evenly distributed bacterial community, including both total bacteria and predicted potential pathogens, associated with coarse particulate matter during autumn.

Chao richness estimates for total bacterial communities ranged from 29 to 1883 across all samples, with seasonal medians of 160 (summer) and 227 (autumn). In terms of particle size fraction: in summer, Chao ranged from 29 to 379 (median = 156) in PM_2.5_ and from 29 to 284 (median = 164) in PM_10_; in autumn, it ranged from 69 to 1026 (median = 164.5) in PM_2.5_ and from 80 to 1883 (median = 1186) in PM_10_. Shannon diversity indices ranged from 0.25 to 6.84, with seasonal medians of 3.62 (summer) and 3.50 (autumn). Stratified by season and fraction: summer PM_2.5_ (0.25–5.46, median = 3.39), summer PM_10_ (2.83–4.30, median = 3.75), autumn PM_2.5_ (1.87–5.46, median = 3.22), and autumn PM_10_ (0.98–6.84, median = 4.25).

Chao richness and Shannon diversity of the predicted potential pathogens ranged from 2 to 348 (mean = 87.86) and from 0.20 to 5.22 (mean = 2.46), respectively. Seasonally, the Chao estimates ranged from 19 to 74 (median = 45.5) in summer and from 18 to 348 (median = 55) in autumn. By particle size fraction: summer PM_2.5_ (4–74, median = 45), summer PM_10_ (31–70, median = 46), autumn PM_2.5_ (18–180, median = 39), and autumn PM_10_ (23–348, median = 229). Shannon diversity ranged from 2.26 to 3.66 (median = 2.69) in summer and from 0.20 to 5.22 (median = 2.28) in autumn. Stratified further: summer PM_2.5_ (0.90–3.66, median = 2.75), summer PM_10_ (2.26–3.24, median = 2.67), autumn PM_2.5_ (0.80–3.95, median = 2.04), and autumn PM_10_ (0.20–5.22, median = 2.45).

### 3.3. The β Diversity of Total Bacteria and Potential Pathogens

**Figure 4:**
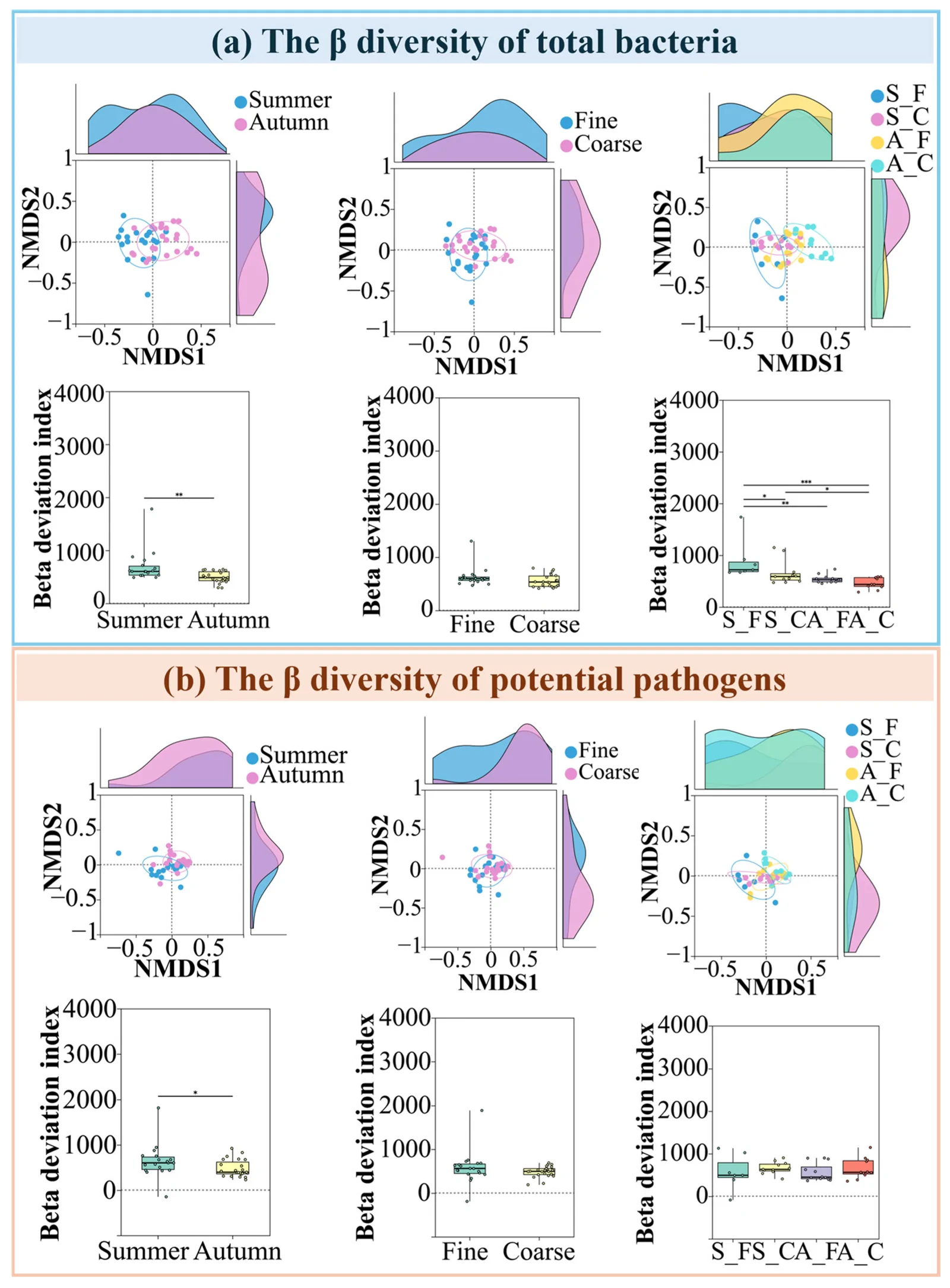
The β diversity of the total bacteria (**a**) and potential pathogens (**b**). (* 0.01 < *p* ≤ 0.05, ** 0.001 < *p* ≤ 0.01, and *** *p* ≤ 0.001).

As shown in Figure 4, β-diversity analysis (Bray–Curtis dissimilarity) revealed significant compositional differentiation in both total bacterial communities (*p* < 0.01) and predicted potential pathogen communities (*p* < 0.05) across sampling groups. Notably, the summer PM_2.5_ samples formed a distinct cluster, significantly divergent from all other groups, including PM_10_ in summer and PM_10_ as well as PM_2.5_ in autumn (*p* < 0.001 for total bacteria), indicating strong seasonal and size-fraction interactions. While the total bacterial communities showed partial overlap between the PM_2.5_ and PM_10_ fractions within each season, potential pathogen communities exhibited greater similarity across particle sizes but retained clear seasonal separation (*p* < 0.05).

**Figure 5:**
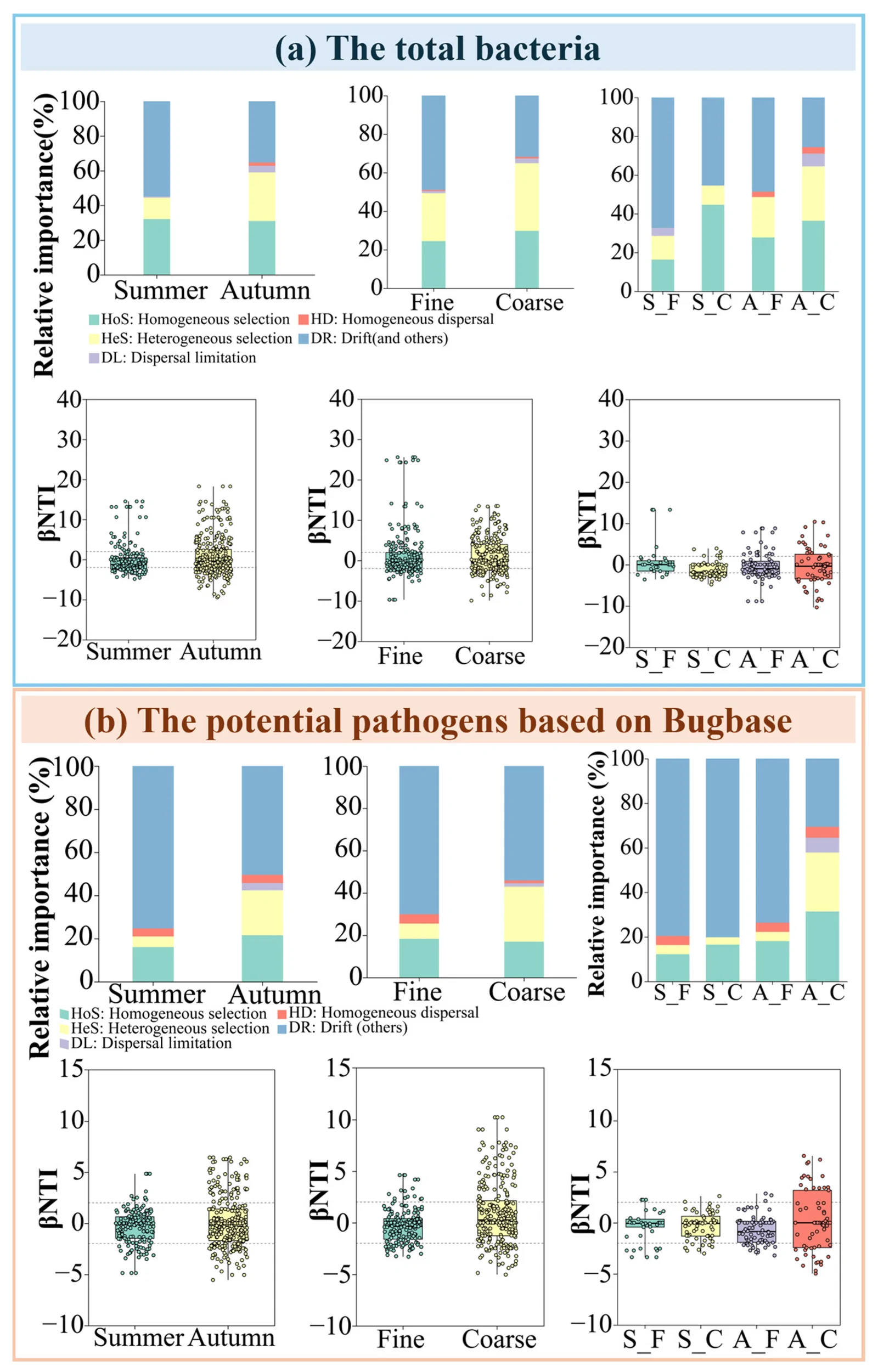
Different ecological processes in the assembly of microbial communities and different patterns of βNTI across season and particle size samples. (**a**) The total bacteria and (**b**) potential pathogens.

Figure 5 presents the inferred ecological assembly processes shaping bacterial communities across seasons and particle size fractions based on βNTI metrics. Homogeneous selection was consistently dominant, accounting for 32.10% of communities in summer and 31.00% in autumn, with no significant seasonal difference. In contrast, heterogeneous selection was markedly elevated in autumn (27.98%) relative to summer (12.35%), and dispersal limitation also increased from 0.62% (summer) to 3.78% (autumn). At the particle size level, PM_10_ (coarse) samples exhibited higher contributions from both heterogeneous selection (35.13% vs. 24.93% in PM_2.5_) and homogeneous selection (29.75% vs. 24.38% in PM_2.5_). Season–size interactions revealed further nuance: autumn PM_10_ communities were characterized by 36.36% homogeneous selection, 28.10% heterogeneous selection, 6.61% dispersal limitation, 3.31% homogeneous dispersal, and 25.62% drift. In contrast, summer PM_2.5_ communities were overwhelmingly governed by stochastic drift (67.35%), accompanied by marked reductions in heterogeneous selection (12.24%), homogeneous selection (16.33%), and dispersal limitation (4.08%).

In the predicted potential pathogen community, stochastic processes, particularly drift, dominated across all samples, while deterministic processes (homogeneous and heterogeneous selection) collectively accounted for a smaller, seasonally variable fraction. Specifically, in summer, drift constituted 75.31% of assembly processes, followed by homogeneous selection (16.05%), heterogeneous selection (4.94%), and homogeneous dispersal (3.70%). In autumn, drift decreased to 50.47%, while homogeneous selection increased to 21.55% and heterogeneous selection rose markedly to 20.79%; dispersal limitation (3.40%) and homogeneous dispersal (3.78%) remained minor contributors. At the particle size level, PM_2.5_ samples exhibited drift (70.08%), homogeneous selection (18.28%), heterogeneous selection (7.20%), and homogeneous dispersal (4.43%). PM_10_ samples showed a distinct profile: drift (54.13%), heterogeneous selection (26.03%), homogeneous selection (16.94%), homogeneous dispersal (1.24%), and dispersal limitation (1.65%). Notably, the autumn PM10 subgroup was the only condition in which all five processes were consistently represented, without substantial decline in any single process, suggesting a more balanced interplay of deterministic and stochastic forces in coarse particulate matter during autumn.

βNTI values for the majority of sample groups fell within the range of −2 to 2, indicating that stochastic (neutral) processes predominated in community assembly. A subset of samples, particularly autumn PM_10_ and summer PM_2.5_, exhibited |βNTI| > 2, suggesting deterministic assembly driven by homogeneous or heterogeneous selection, respectively.

## 4. Discussion

Airborne bacterial communities in Qingdao have been previously characterized; however, this study advances prior work by providing a systematic, quantitative assessment of both the total airborne bacterial abundance and the taxonomic–functional potential for pathogenicity across diverse particulate matter (PM) fractions and seasonal conditions. A central finding is the persistent dominance of *Staphylococcus* in all sampled aerosols, irrespective of seasonality or PM size fraction. *Staphylococcus* was reported to exhibit significant enrichment at inland sampling sites under northerly air mass advection [24]. It originates predominantly from anthropogenic emissions and natural environmental reservoirs. Environmental reservoirs, including soil, freshwater, and marine sediments, serve as emission sources that further augment atmospheric *Staphylococcus* loads [35]. As commensal constituents of human skin and gut microbiota, these *staphylococci* typically remain nonpathogenic in healthy individuals; however, they pose substantial infection risks under conditions of microbial dysbiosis, host immunosuppression, or breach of anatomical barriers, particularly in vulnerable populations including hospitalized patients and those with indwelling medical devices [35,36]. Importantly, the recurrent detection of *Staphylococcus* in urban aerosols beyond Qingdao, specifically as a putative pathogen in autumn-collected particulate matter (PM) from the Belgrade metropolitan area [37], reinforces its cross-regional relevance and supports its consideration as a high-priority microbial indicator for integrated air quality and public health risk assessment.

In Qingdao, a prior study reported the consistent detection of *Pseudomonas* (average relative abundance = 58.37%), *Ralstonia* (average relative abundance = 4.17%), *Methyloversatilis* (average relative abundance = 4.73%), and *Bacteroides* (average relative abundance = 2.32%) across all sampled air filters [25], highlighting site-specific microbial signatures. Consistent with this, Xie et al. (2021) synthesized evidence from multiple urban studies and demonstrated that airborne bacterial community composition is strongly shaped by local environmental and anthropogenic drivers, resulting in city-specific taxonomic profiles [38]. For instance, *Pseudomonas*, *Delftia*, *Serratia*, and *Acinetobacter* dominated in Urumqi [39]; *Acinetobacter*, *Massillia*, and *Xanthomonadaceae* were most abundant in Changsha [40]; while *Streptophyta*, *Kocuria*, *Paracoccus*, *Sphingomonas*, and *Rubellimicrobium* collectively accounted for the highest relative abundances in Beijing [41].

Even within a single city, microenvironmental variation, particularly diel cycles, exerted a measurable influence on airborne bacterial community structure. In Hangzhou, diurnal airborne communities were consistently dominated by Cyanobacteria (mean relative abundance = 30%; range: 25–36% across sampling timepoints) and Alphaproteobacteria (26%), followed by Firmicutes (9%), Bacteroidetes (8%), Actinobacteria (6%), and Betaproteobacteria (6%). Notably, 14 out of 421 genera were detected in >90% of diurnal samples, whereas 37 out of 409 genera exhibited comparable ubiquity across nocturnal samples, suggesting distinct core assemblages shaped by day–night environmental gradients. [31]. Beyond China, distinct patterns emerge: *Pseudomonas*, *Herbaspirillum*, and *Bacillus* dominate in Thessaloniki [42], while *Kocuria*, *Arthrobacter*, *Sphingomonas*, and *Pantoea* are consistently abundant in Madrid [43]. Collectively, these geographically resolved profiles underscore the value of multi-city comparative studies in building a robust, spatially explicit database of airborne bacterial distribution and in identifying both locally persistent taxa and regionally conserved functional guilds.

Regarding bacterial α-diversity, a previous study reported higher diversity in PM_10_ than in PM_2.5_, with several genera detected exclusively in coarse particles [44]. In this study, coarse particles exhibited marginally higher Chao and Shannon indices than fine particles, yet this difference was not statistically significant across the full dataset. A significant increase in both indices was observed only in autumn PM_10_ samples relative to all other season–size combinations, indicating a seasonally intensified size-dependent diversification effect. For the subset of predicted potential pathogens, no significant differences in Chao or Shannon indices were found between fine and coarse particles. Likewise, no significant seasonal variation in α-diversity was detected across the summer and autumn groups for either total bacteria or potential pathogens. Collectively, these findings align with previous reports indicating that airborne bacterial communities are stratified by aerodynamic particle size, with elevated taxonomic diversity concentrated in particles near 2.5 μm and 10 μm [45].

The airborne bacterial community data generated in this study advance the current understanding of the taxonomic composition, spatiotemporal dynamics, and size-resolved distribution of outdoor airborne bacteria in coastal urban environments. These findings have direct implications for indoor air quality management, particularly given that outdoor-derived microbes are readily transported indoors and can colonize built-environment reservoirs. For instance, our prior work demonstrated that indoor air conditioning filters consistently harbor abundant outdoor-associated taxa, including Cyanobacteria, suggesting a continuous infiltration pathway from ambient air into indoor spaces [46,47,48]. Characterizing outdoor airborne microbial communities is essential for understanding their contribution to indoor air microbiomes, as ambient air serves as a primary source of indoor bioaerosols through infiltration, ventilation, and natural air exchange [21].

In summary, this study advances the understanding of airborne bacterial communities and potential pathogens. Several limitations warrant consideration for future work. First, the culture-independent approach employed here does not distinguish viable from non-viable bacteria, a key constraint given the inherent difficulty of culturing many environmental microorganisms. Integrating complementary culture-based methods could therefore enhance viability assessment and functional characterization. Second, taxonomic resolution for many potential pathogenic bacteria was limited to the genus level; deeper identification (e.g., species-level) using full-length 16S rRNA gene sequencing or metagenomic binning is recommended to improve pathogen risk assessment.

## 5. Conclusions

The airborne bacterial community exhibited pronounced seasonal variation in both taxonomic composition and diversity structure. *Staphylococcus*, *Lactobacillus*, *Escherichia*–*Shigella*, and *Klebsiella* were consistently dominant across all samples. LEfSe analysis identified *Staphylococcus* as significantly enriched in autumn, supporting its utility as a robust seasonal biomarker. The overall community composition differed significantly between summer and autumn. The Chao and Shannon alpha diversity indices were consistently higher in coarse particulate matter than in fine particles across seasons; however, this size-associated difference reached statistical significance only for the autumn PM_10_ samples, indicating a seasonally amplified effect of particle size on microbial richness and evenness. It provides foundational data on airborne potential pathogenic bacteria to support future quantitative microbial risk assessments in coastal urban environments, thereby enabling integrated evaluations of the risks to ecosystem integrity and public health, and delivering valuable bioinformatic resources for advancing microbial ecology research.

## Data Availability

The data presented in this study are not publicly available due to privacy and available upon request from the corresponding authors.

## Supplementary Materials

The following supporting information can be downloaded at: https://www.mdpi.com/article/10.3390/microorganisms14091924/s1, Figure S1: The relative abundance of airborne bacteria at species level; Table S1: The details of collected samples; Table S2: The groups of collected samples.
