# Supplementary material for: Seasonal Variation of Airborne Bacteria and Potential Pathogens Adhere to Fine and Coarse Particles in a Coastal City
Source: Microorganisms. 2026 Aug 31;14(9):1924. doi: 10.3390/microorganisms14091924 (PMC13609889; doi:10.3390/microorganisms14091924)
Supplement: Supplementary file 1 [file microorganisms-14-01924-s001.zip › microorganisms-4486730-supplementary.pdf]

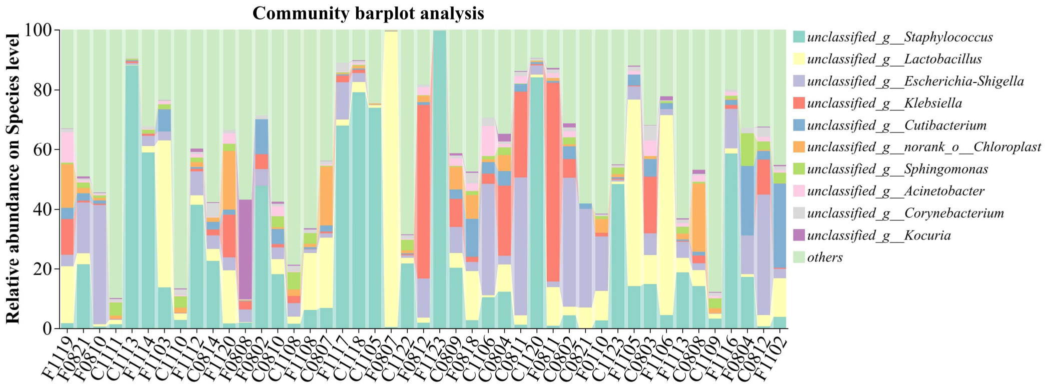

Figure S1. The relative abundance of airborne bacteria at species level.

Table S1. The details of collected samples.

Sample ID	Date	Sample	Temperature ºC	Sample ID	Date	Sample	Temperature ºC
F0802	8.2	$PM_{2.5}$	37.6	F1102	11.2	$PM_{2.5}$	20.9
F0804	8.4	$PM_{2.5}$	37.9	F1103	11.3	$PM_{2.5}$	21.4
F0807	8.7	$PM_{2.5}$	38.5	F1105	11.5	$PM_{2.5}$	19.7
F0808	8.8	$PM_{2.5}$	32.9	F1106	11.6	$PM_{2.5}$	21.8
F0810	8.10	$PM_{2.5}$	33.5	F1108	11.8	$PM_{2.5}$	9
F0811	8.11	$PM_{2.5}$	35.6	F1112	11.12	$PM_{2.5}$	15.7
F0812	8.12	$PM_{2.5}$	28.3	F1113	11.13	$PM_{2.5}$	18
F0818	8.18	$PM_{2.5}$	39.2	F1114	11.14	$PM_{2.5}$	18.7
F0821	8.21	$PM_{2.5}$	34.2	F1116	11.16	$PM_{2.5}$	17.4
C0802	8.2	PM_{10}	37.6	F1117	11.17	$PM_{2.5}$	19.3
C0803	8.3	PM_{10}	35.7	F1119	11.19	$PM_{2.5}$	18
C0804	8.4	PM_{10}	36.3	F1120	11.20	$PM_{2.5}$	18.8
C0807	8.7	PM_{10}	34.9	F1123	11.23	$PM_{2.5}$	11.7
C0808	8.8	PM_{10}	37.7	C1105	11.5	PM_{10}	20.1
C0809	8.9	PM_{10}	37.9	C1106	11.6	PM_{10}	21.4
C0810	8.10	PM_{10}	36.2	C1108	11.8	PM_{10}	10.3
C0811	8.11	PM_{10}	32.4	C1109	11.9	PM_{10}	11.7
C0812	8.12	PM_{10}	27.1	C1110	11.10	PM_{10}	13.3
C0814	8.14	PM_{10}	32.8	C1111	11.11	PM_{10}	13.2
C0821	8.21	PM_{10}	32.7	C1113	11.13	PM_{10}	17.6
				C1118	11.18	PM_{10}	20.5
				C1120	11.20	PM_{10}	18.8
				C1122	11.22	PM_{10}	9.2
				C1123	11.23	PM_{10}	11.9

Table S2. The groups of collected samples.

Fine	Coarse	Summer	Autumn	S_F	S_C	A_F	A_C
F0802	C0802	F0802	F1102	F0802	C0802	F1102	C1105
F0804	C0803	F0804	F1103	F0804	C0803	F1103	C1106
F0807	C0804	F0807	F1105	F0807	C0804	F1105	C1108
F0808	C0807	F0808	F1106	F0808	C0807	F1106	C1109
F0810	C0808	F0810	F1108	F0810	C0808	F1108	C1110
F0811	C0809	F0811	F1112	F0811	C0809	F1112	C1111
F0812	C0810	F0812	F1113	F0812	C0810	F1113	C1113
F0818	C0811	F0818	F1114	F0818	C0811	F1114	C1118
F0821	C0812	F0821	F1116	F0821	C0812	F1116	C1120
F1102	C0814	C0802	F1117		C0814	F1117	C1122
F1103	C0821	C0803	F1119		C0821	F1119	C1123
F1105	C1105	C0804	F1120			F1120	
F1106	C1106	C0807	F1123			F1123	
F1108	C1108	C0808	C1105				
F1112	C1109	C0809	C1106				
F1113	C1110	C0810	C1108				
F1114	C1111	C0811	C1109				
F1116	C1113	C0812	C1110				
F1117	C1118	C0814	C1111				
F1119	C1120	C0821	C1113				
F1120	C1122		C1118				
F1123	C1123		C1120				
			C1122				
			C1123				
